# The effects of temperature on *Bosmina longirostris* susceptibility to microcystin-LR acute toxicity

**DOI:** 10.1371/journal.pone.0219342

**Published:** 2019-07-09

**Authors:** Madison C. Lamb, David G. Kimmel, Erin K. Field

**Affiliations:** 1 Department of Biology, East Carolina University, Greenville, North Carolina, United States of America; 2 US National Oceanic and Atmospheric Administration, Alaska Fisheries Science Center, Seattle, Washington, United States of America; University of Connecticut, UNITED STATES

## Abstract

Harmful algal blooms are an ongoing threat to many aquatic systems throughout the world. In the Chowan River, North Carolina, the frequency of toxin producing *Microcystis aeruginosa* blooms has increased since 1975 along with an average 0.71°C rise in water temperature. The combined effect of microcystin-LR toxin and rising temperatures on a dominant zooplankter in the system, *Bosmina longirostris*, was the focus of this study. Laboratory studies were conducted to determine how microcystin-LR, produced from *M*. *aeruginosa* blooms, affected *B*. *longirostris* mortality under different temperature regimes. At 25°C, the LC_50_ for *B*. *longirostris* was 26.3 μg L^-1^ suggesting that *B*. *longirostris* can survive typical current bloom microcystin-LR concentrations ranging 0.1μg L^-1^ to 2.0 μg L^-1^, but would be susceptible to higher concentrations they may be periodically exposed to. Mortality was assessed at a constant microcystin-LR concentration of 26.3 μg L^-1^ over 15–35°C, and it was found that *B*. *longirostris* mortality increased at higher temperatures. *B*. *longirostris* mortality increased approximately 18% due to microcystin-LR alone over 2°C between 25°C and 27°C when exposed to the LC_50_ concentration. The increased prevalence of toxic *M*. *aeruginosa* blooms and increasing temperatures due to climate change may reduce *B*. *longirostris* populations, potentially affecting larval fish and fisheries in the Chowan River, North Carolina.

## Introduction

Harmful algal blooms have been increasing worldwide and continue to be a major management concern [1–3]. A consensus among scientists revealed that the persistence and expansion of harmful algal blooms was cause for further empirical studies [4]. Climate change remains one of the most important environmental stressors, leading to increased harmful algal blooms and contributing to higher global temperatures [5, 6]. Warmer temperatures often result in a positive feedback cycle for harmful algal blooms [7]. Increasing sea surface temperatures have specifically led to concern of changes in cyanobacteria harmful algal bloom taxa and increased frequency, worldwide [8, 9].

Harmful algal blooms are known to occur under highly eutrophic conditions in lower salinity waters [10]. These conditions are currently present in the Chowan River, a coastal river in North Carolina. Thirty years ago, blooms of the cyanobacterium *Microcystis aeruginosa* were common in the Chowan River [11]; however, a combination of the phosphorus detergents ban in the early 1980s and the shutdown of a local fertilizer plant [12] resulted in a reduction of blooms until recently. Over the last decade, *M*. *aeruginosa* blooms have been reoccurring in the Chowan River [13, 14]. In August of 2013, a *M*. *aeruginosa* bloom exhibited a particularly high microcystin-LR toxin level of 68 μg L^-1^, which is up to 34 times the typical toxin concentrations in the Chowan River of 0.1 μg L^-1^ to 2.0 μg L^-1^ [13]. Blooms are therefore increasing in frequency and occasionally high concentrations making them more dangerous and problematic.

Microcystin-LR toxins are released into surrounding waters when *M*. *aeruginosa* cells die and cell lysis occurs [15], exposing the toxins to the entire aquatic food web. At the planktonic level, zooplankton serve as one of the major trophic links between microcystin-LR and the rest of the food web. Zooplankton can be exposed to microcystin-LR that has dissolved directly in the water column or via ingestion of *M*. *aeruginosa* cells. Microcystin-LR can cause direct mortality [16–18], eliminating susceptible zooplankton from the food web, and thereby reduce the prey available to larval and juvenile fish. *Bosmina longirostris* is a ubiquitous species of herbivorous, freshwater zooplankter that is dominant in abundance within the Chowan River system in the spring months of April through June [19]. *B*. *longirostris* does not preferentially avoid toxic *M*. *aeruginosa* cells [18], which suggests that they may be resistant to microcystin-LR and survive in its presence [20]. *B*. *longirostris* is found in the diets of anadromous fish species present along the Southeast United States’ coast, such as American shad (*Alosa sapidissima*) and both species of river herring Alewife (*A*. *pseudoharengus*) and Blueback herring (*A*. *aestivalis*) [21–23]. Within the Southeast, American shad and river herring fish stocks are considered depleted [24], and therefore a diet composed of *B*. *longirostris* is particularly important.

Microcystin-LR can be prevalent for up to two weeks after a bloom and may be found in higher concentrations at higher temperatures [25]. Some species of small zooplankton have also been correlated with microcystin concentrations [26]. Since zooplankton biological rates are temperature dependent and grazing rates increase with temperature [27, 28], microcystin-LR poses a threat to zooplankton and aquatic food webs. Global sea surface temperatures have risen approximately 0.6±0.3°C since 1854 [29]. The Chowan River, specifically, has warmed by 0.71°C since 1975 [11, 13].

Temperature changes due to climate change could increase the susceptibility of zooplankton to toxins produced by harmful algal blooms. The reoccurring frequency of *M*. *aeruginosa* blooms in the Chowan River suggests the need for research to determine how microcystin-LR toxins at an individual level impact zooplankton under increasing temperatures. Several other studies have looked at the effect of temperature and toxic *M*. *aeruginosa* cells on zooplankton mortality. Most studies tend to focus on the Genus *Daphnia* [17, 30], often overlooking small-bodied cladocerans, like *B*. *longirostris*. Hietala, Laurén-Määttä, and Walls exposed *Daphnia pulex*, a larger-bodied cladoceran, to microcystin-LR at two different temperatures: 19°C and 24°C [31]. *D*. *pulex* showed increased mortality to microcystin-LR at higher temperatures [31]. This pattern was also seen in rotifers where increasing temperatures increased the sensitivity of rotifers to *M*. *aeruginosa* cells [32]. Jiang et al. previously examined the effect of toxic *M*. *aeruginosa* cells and temperature on *B*. *longirostris* separately and found that *B*. *longirostris* experienced a greater mortality at higher temperatures when exposed to *M*. *aeruginosa* cells [33]. No studies; however, have effectively evaluated the combined effect of temperature and specifically microcystin-LR at a set concentration on *B*. *longirostris*. The purpose of this study was to investigate the effect of temperature on *B*. *longirostris* susceptibility to microcystin-LR at a specific concentration as it has important effects on the aquatic food web and determine if increasing temperature during exposure to microcystin-LR would result in increased mortality.

## Materials and methods

### Organism collection

*B*. *longirostris* were collected from Catherine’s Creek, a tributary of the coastal Chowan River in North Carolina (N36.314706, W76.670449) in May and June of 2016 and April of 2017 using a 0.25 m diameter, 200 μm mesh plankton net towed via kayak. Collection of organisms did not require permission or involve endangered animals. At each sampling time, background microcystin-LR concentrations were below the detectable limit of 0.15 ng mL^-1^ via Enzyme-linked immunosorbent assay (ELISA) analysis conducted by Greenwater Laboratories in Palatka, FL. *B*. *longirostris* were stored in 4 L plastic containers on ice during transport from the field to East Carolina University. Organisms were fed unicellular Instant Algae *Isochrysis* 1800 (stock density 3.9 billion cells mL^-1^) within unfiltered Chowan River water medium and kept at 4°C in the dark until experimental use. Organisms were acclimated to room temperature during experimental set up (approximately 6 hours, see details below) in order to minimize stress before experiments began. Organisms used in experiments were actively swimming, healthy adults, approximately 0.5 mm in size. All collected *B*. *longirostris* were used in toxicity and temperature experiments within one week of collection from Catherine’s Creek, NC in an effort to minimize long-term stress effects.

Chowan River water for toxicity experiments was collected simultaneously with organism collection using 10 L containers. Water for experiments was vacuum-filtered immediately using 45 mm diameter, 0.7 μm Whatman glass-fiber filters. Filtered Chowan River water was then stored in the dark at 4°C until experimental use.

### Microcystin-LR LC_50_ for *B*. *longirostris*

In order to determine the LC_50_ concentration for *B*. *longirostris*, a dose-response curve experiment was set up with a range of microcystin-LR concentrations: 0, 0.005, 0.05, 0.5, 5.0, 50, and 100 μg L^-1^ Microcystin-LR (Sigma-Aldrich). Each microcystin-LR concentration contained 10 *B*. *longirostris* individuals and 10 μL of Instant Algae Isochrysis 1800 in 100 mL glass containers of filtered Chowan River water. *B*. *longirostris* for this experiment were collected from the Catherine’s Creek, Chowan River in May of 2016. Organisms were separated from stock bottles and acclimated to 25°C for six hours (one hour for acclimation and five hours to set up experiment and add organisms to treatment vials), prior to addition of microcystin-LR. Triplicate bottles were set up for each microcystin-LR concentration and experiments were conducted for 48 hours at 25°C to determine the LC_50_ concentration. An exposure time of 48 hours was chosen because it is the most commonly used exposure period [30, 31, 34, 35]. The temperature of 25°C was selected because it is the median between the study ranges of 15–35°C. The LC_50_ is defined here as the lethal concentration that is fatal to 50% of a population. After 48 hours, *B*. *longirostris* were filtered out using a 60 μm mesh sieve and assessed for mortality using microscopy by counting the number of live and dead organisms. In this instance, death was determined by complete cease of all movement. This entire experiment was repeated twice.

It is important to note that the microcystin-LR stock used in the dose-response study was created from dissolving 1 mg of microcystin-LR vial in 1 mL of 100% ethanol (per for the manufacturer’s recommendation) in order to make the microcystin-LR more soluble in Chowan River water. Then, approximately 46.66 μL of the microcystin-LR stock vial (1 mg mL^-1^ in 100% ethanol) was diluted with 100 mL of filtered Chowan River water to create a microcystin-LR stock solution to be used in experimentation. This new microcystin-LR stock solution was then used to create triplicates of seven different microcystin concentrations, as mentioned before, by adding calculated amounts of the stock solution into respective 100 mL jars filled with filtered Chowan River water. At the lowest microcystin-LR concentration of 0.005 μg L^-1^, there was approximately 0.0005 μL of ethanol remaining in the 100 mL jar. Comparatively, at the highest microcystin-LR concentration of 100 μg L^-1^, there was approximately 10 μL of ethanol remaining. At the LC_50_ concentration there was approximately 2.6 μL of ethanol remaining, contributing to approximately 2% mortality. The remaining amount of ethanol was not notably toxic to organisms based on a preliminary experiment conducted and did not affect the overall statistical significance of the results of microcystin-LR toxicity to *B*. *longirostris* for all experiments, nor did it affect calculation of the range of the LC_50_.

All statistical analyses were conducted using SAS University Edition. Prior to statistical tests, homoscedasticity (via Levene’s Test and Welch’s ANOVA) and normality were assessed. Assumptions of the Analysis of Covariance (ANCOVA) and Analysis of Variance (ANOVA) were met. A single factor ANOVA followed by a Fisher’s LSD pairwise comparison *a posteriori* test was used to determine which microcystin-LR treatments were statistically different from each other. A dose-response curve was initially used to determine the LC_50_ at the value in which 50% of *B*. *longirostris* mortality was reached. This LC_50_ was later confirmed by the probit analysis method described by Finney [36]: a common method used in the literature to calculate LC_50_ values from toxicology studies [30, 34, 35]. The probit analysis method transforms sigmoid dose-response data into a straight line in which a linear regression can be fit to calculate the LC_50_ [36]. Data from this experiment exhibited a normal distribution and therefore a probit analysis was used rather than a logit analysis, which assumes a non-normal distribution. Percent mortalities were transformed into empirical probits corrected for mortality, using Table 3.2 described in Finney [36], from the log-transformed controls and microcystin-LR concentrations.

### Effect of temperature on microcystin-LR toxicity

*B*. *longirostris* mortality was then assessed at the LC_50_ (26.3 μg L^-1^) over a range of temperatures: 25°C, 27°C, 30°C, 32°C, and 35°C during a 48-hour exposure to determine the effect of varying temperature at a constant microcystin-LR concentration on *B*. *longirostris*. Each temperature treatment was maintained within ±0.1°C using an incubator. Each temperature treatment contained 10 *B*. *longirostris*, 10 μL of Instant Algae Isochrysis 1800, and 26.3 μg L^-1^ of microcystin-LR in 100 mL glass containers of filtered Chowan River water. Control treatments were set up for each temperature treatment, which contained no microcystin-LR toxin. *B*. *longirostris* for this experiment were collected in June of 2016. Triplicate bottles were set up for each temperature treatment with the entire experiment repeated three times. After 48 hours, *B*. *longirostris* were filtered out using a 60 μm mesh sieve and assessed for mortality using microscopy by counting the number of live and dead organisms, in the same manner as before.

This entire experiment was then again repeated three times using a lower range of temperatures within *B*. *longirostris’* range of thermal tolerance [37]: 15°C, 17°C, 20°C, 22°C, and 25°C. *B*. *longirostris* used in this temperature range study were collected in April of 2017 and the same experimental setup as the higher temperature range was followed. An ANCOVA then was conducted for temperature experiments to determine the significance of the interaction between microcystin-LR and temperature on *B*. *longirostris* mortality.

### Microcystin-LR concentration analysis

Concentrations were measured to determine if the microcystin-LR concentration changed due to degradation within the 48-hour exposure period during the temperature experiment. Evaluation of microcystin-LR concentration (LC_50_) used for the temperature experiments was conducted using an ELISA analysis at Greenwater Laboratories in Palatka, Florida. One sample from the 27°C temperature treatment and two samples from the 25°C treatments were analyzed for microcystin-LR content to determine the treatment effects on the LC_50_ concentration.

## Results

### Microcystin-LR LC_50_ for *B*. *longirostris*

**Fig 1:**
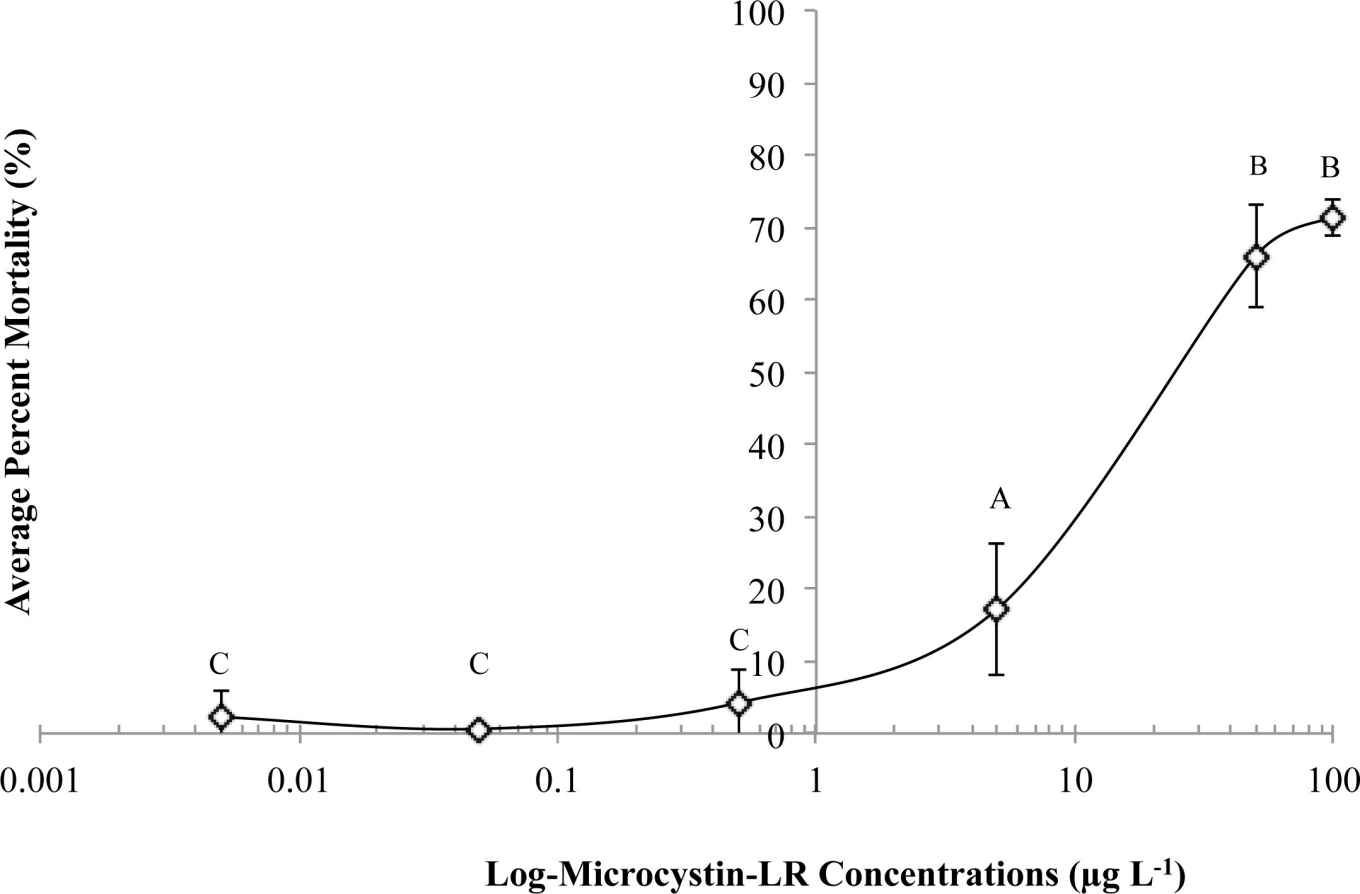
LC_50_ dose-response curve for *B*. *longirostris*. Average percent mortality of *B*. *longirostris* collected in May 2016 following a 48-hr exposure at 25°C to a range of microcystin-LR concentrations. Data corrected for control percent mortality. Error bars represent standard deviation. N = 6. Different letters indicate significant difference (p-value<0.05, Fisher’s LSD). One-way ANOVA: *p*<0.0001. *F*_*5*,*30*_ = 139.96. *R*^*2*^ = 0.96.

**Fig 2:**
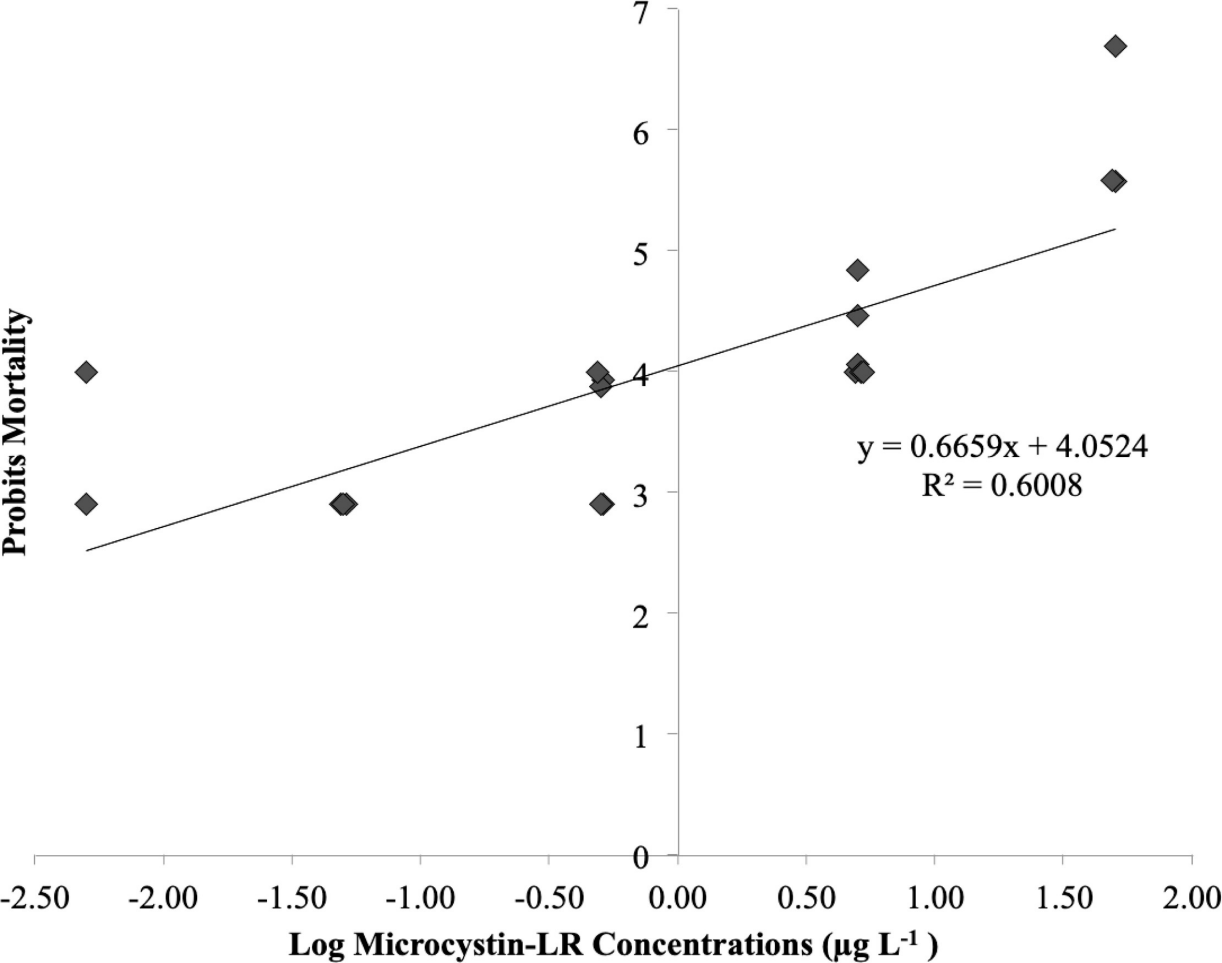
Probit analysis of the effect of microcystin-LR on *B*. *longirostris* mortality. Percent mortality converted to empirical probits versus log of the microcystin-LR concentrations [36]. Log-LC_50_ = 1.42, based on linear regression with 100 μg L^-1^ concentration treatment omitted: *y* = 0.6666x+4.0531. *R*^*2*^ = 0.602. LC_50_ = 26.3±4.6 μg L^-1^.

Results from this experiment showed that *B*. *longirostris* mortality increased with increasing microcystin-LR concentrations (Fig 1). The LC_50_ for *B*. *longirostris* exposure to microcystin-LR was determined to be 26.3±6.4 μg L^-1^ for 48 hours at 25°C using Finney’s [36] probit analysis method (Fig 2). The difference in percent mortality between treatments was significantly explained by the change in microcystin-LR concentration (Fig 1) (ANOVA, *F*_*5*,*30*_ = 139.96, *p*<0.0001). Microcystin-LR concentrations of 0.5, 5.0, and 50 μg L^-1^ were found to be statistically different from each other, with the LC_50_ concentration of 26.3 μg L^-1^ calculated in between 5.0 μg L^-1^ and 50 μg L^-1^ (Fig 1) (Fisher’s LSD, *p*<0.001). A two-tailed, two-sample t-test revealed that the LC_50_ of 26.3 μg L^-1^ was significantly higher than typical concentrations of microcystin-LR in the Chowan River for 2012: 0.1 μg L^-1^ to 2.0 μg L^-1^ (*p*<0.05) [13]. Results from the ELISA analysis of one 27°C sample and two 25°C samples, one replicate from the higher temperature range experiment (25°C-35°C) and one replicate from the lower range experiment (15°C-25°C) revealed no change in microcystin-LR concentration over the course of 48-hour treatments due to evaporation and confirmed that the microcystin-LR concentration used for all studies was consistent within the range of the LC_50_ of 26.3±6.4 μg L^-1^.

### Effect of temperature on *B*. *longirostris* susceptibility to microcystin-LR

**Fig 3:**
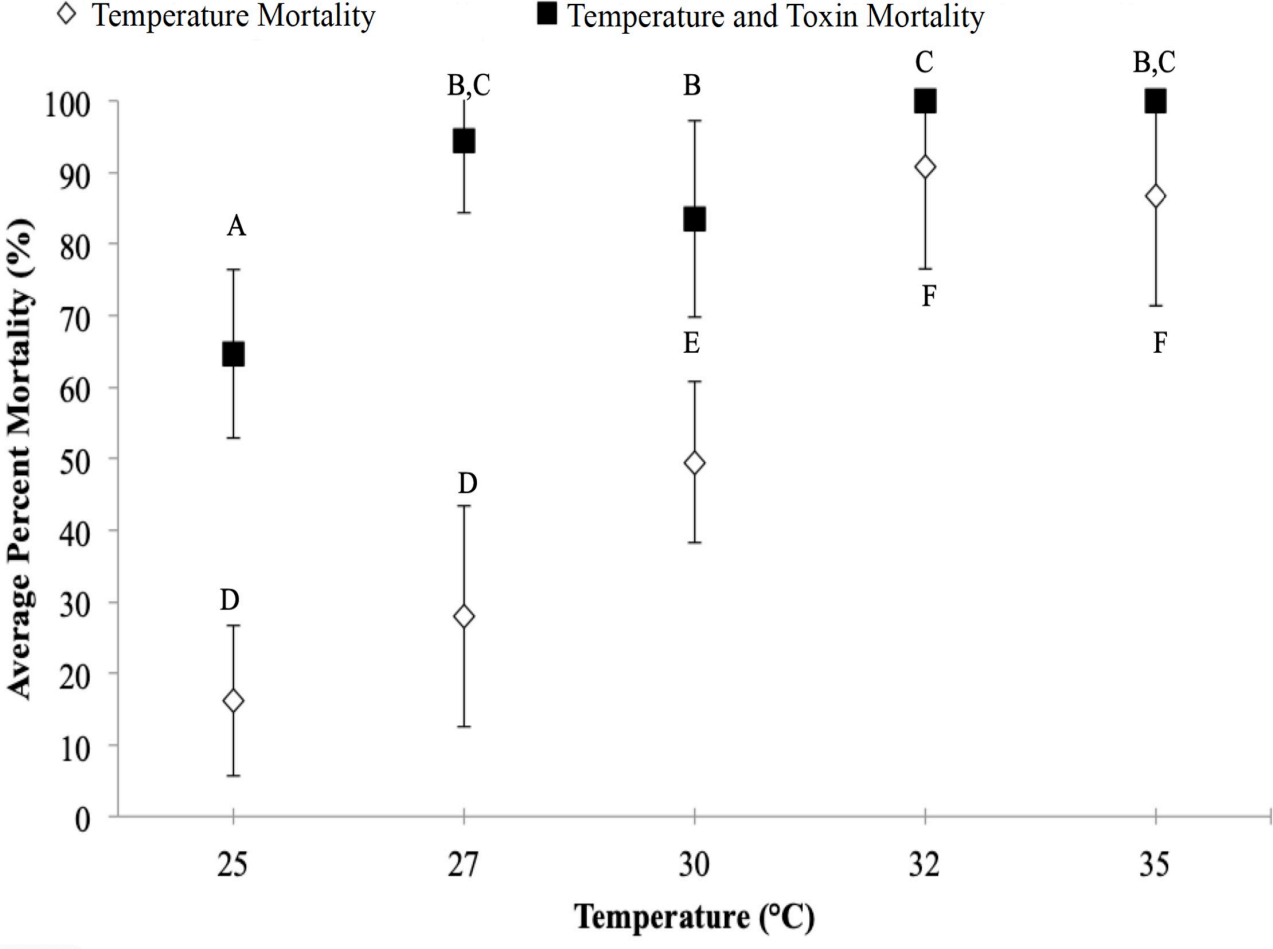
Temperature and microcystin-LR exposure from 25°C-35°C. Average percent mortality of *B*. *longirostris* collected in June 2016 over a range of temperatures when exposed to the LC_50_ of 26.3 μg L^-1^ microcystin-LR for 48-hrs. Temperature control treatments did not contain microcystin-LR. Error bars represent standard deviation. N = 9. Different letters indicate significant difference (p-value<0.05, Fisher’s LSD). ANCOVA: *p* <0.0001. *R*^*2*^ = 0.80. *F*_*1*,*1*_ = 23.18. Temperature one-way ANOVA: *R*^*2*^ = 0.84. *p*-value <0.0001. *F*_*4*,*30*_ = 40.23. Temperature plus Toxin one-way ANOVA: *R*^*2*^ = 0.68. *p*-value <0.0001. *F*_*4*,*30*_ = 15.92.

The typical temperature range that *B*. *longirostris* may experience in the Chowan River (15–35°C) was tested. Increasing temperatures resulted in a significant increase in temperature plus toxin percent mortality (combined mortalities of microcystin-LR and temperature) when *B*. *longirostris* collected in June 2016 were exposed to microcystin-LR at 26.3 μg L^-1^ from 25–35°C (64.6%-100%) (Fig 3, ANOVA: *R*^*2*^ = 0.68, *F*_*4*,*30*_ = 15.92, *p*<0.0001). There was a 30% increase in temperature plus toxin mortality specifically between 25°C and 27°C. An analysis of covariance showed that the presence of microcystin-LR significantly affected the mortality of *B*. *longirostris* between 25°C and 27°C (ANCOVA, *F*_*1*,*68*_ = 23.18, *p* <0.0001).

Temperature increased the susceptibility of *B*. *longirostris* to microcystin-LR. There was an 18% increase in mortality between 25°C and 27°C due to microcystin-LR, alone, calculated from the difference between the average increase in mortality between the temperature plus toxin and the temperature control for 25°C to 27°C. This 18% increase in mortality over a 2°C increase in temperature shows that, while the concentration of microcystin-LR remained the same at 26.3 μg L^-1^, *B*. *longirostris* became more susceptible to microcystin-LR. Combined temperature and microcystin-LR exposure led to a 94% temperature plus toxin mortality at 27°C (Fig 3). Above 30°C, 100% temperature plus toxin mortality was observed.

**Fig 4:**
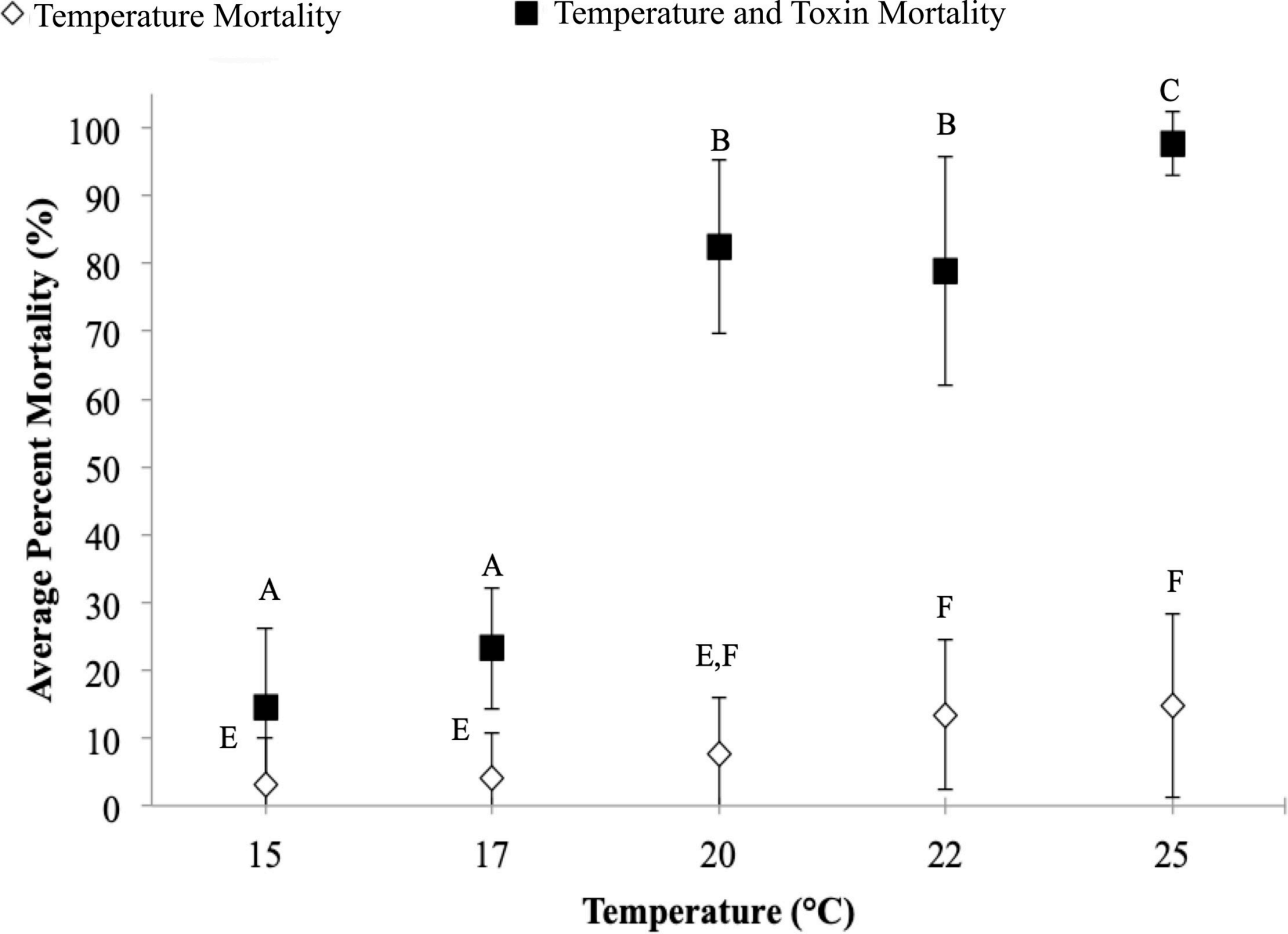
Temperature and microcystin-LR exposure from 15°C-25°C. Average percent mortality of *B*. *longirostris* collected in April 2017 over a range of temperatures when exposed to the LC_50_ of 26.3 μg L^-1^ microcystin-LR for 48-hrs. Temperature control treatments were without microcystin-LR. Error bars represent standard deviation. N = 9. Different letters indicate significant difference (*p*<0.05, Fisher’s LSD). ANCOVA: *p*<0.0001. *R*^*2*^ = 0.88. *F*_*1*,*1*_ = 92.48. Temperature one-way ANOVA: *R*^*2*^ = 0.21. *p*<0.05. *F*_*4*,*44*_ = 2.27. Temperature plus Toxin one-way ANOVA: *R*^*2*^ = 0.90. *p*<0.0001. *F*_*4*,*41*_ = 85.37.

When the experiment was repeated in April 2017 over a lower temperature range (15°C-25°C) within *B*. *longirostris’* lower thermal tolerance, it was seen that temperature plus toxin mortality also increased significantly with temperature, indicating this trend was not limited to the higher end of the tolerance range (Fig 4) (ANOVA, *F*_*4*,*37*_ = 85.37, *p*<0.0001). Temperature plus toxin mortality increased by 83% over the 10°C range from 15°C to 25°C (Fig 4). Between 15°C and 17°C, temperature plus toxin mortality averaged 18.8%, but then increased to 82.5% over a 3°C range from 17°C to 20°C (Fig 4). An analysis of covariance confirmed that the presence of microcystin-LR significantly affected the mortality of *B*. *longirostris* at each lower temperature treatment (ANCOVA, *F*_*1*,*83*_ = 92.48, *p* <0.0001). This again demonstrated that while the concentration of 26.3 μg L^-1^ remained constant, the susceptibility of the April 2017 *B*. *longirostris* population to microcystin-LR increased with temperature over this range.

## Discussion

This study found that *B*. *longirostris* demonstrated an LC_50_ of 26.3 μg L^-1^ when exposed to microcystin-LR at 25°C. Based on an LC50 of 26.3 μg L^-1^, *B*. *longirostris* populations would be able to survive typical current microcystin-LR concentrations in the Chowan River and surrounding Albemarle Sound, which range from less than 0.1 μμg L^-1^ to 2.0 μg L^-1^ [13]. Between the concentrations of 0.1–2.0 μg L^-1^, *B*. *longirostris* experiences a mortality of 3.6% to 7.1%, compared to the 50% mortality at the LC50 (26.3 μg L^-1^) (Fig 1). Therefore, approximately 93% of *B*. *longirostris* survive current microcystin-LR concentrations in the Chowan River. However, high microcystin-LR concentrations may negatively affect *B*. *longirostris* population survivorship. The 2013 bloom, for example, which had a microcystin-LR concentration of 68 μg L^-1^ [13], could have killed a high proportion of the population of *B*. *longirostris* (approximately 75% of the population based on the conditions tested here) (Fig 1). Additionally, sub-lethal effects of microcystin-LR to *B*. *longirostris* include: inhibited filtering rate [38], decreased growth rate [33], and decreased or non-existent reproduction [20]. Therefore, if blooms of this concentration were to increase in frequency in the future, then the *B*. *longirostris* populations may experience large population size changes. Monitoring these population sizes after high concentration blooms will help link these results to *in situ* conditions in the Chowan River and account for other environmental factors that may play a role.

In comparison to other zooplankton, *B*. *longirostris* were found to have the lowest resistance to microcystin-LR, indicating that *B*. *longirostris* may be more susceptible to the effects of microcystin-LR than other species (Table 1). Larger-bodied cladocerans, like *D*. *pulex*, have an LC50 of 9600 μg L^-1^ when exposed to microcystin-LR [30]. Other cladocerans, like *Daphnella hyalina* and *Daphnia pulicaria*, appear to have the highest LC50 values and demonstrate higher resistance to microcystin-LR compared to *B*. *longirostris* [30]. Copepods are another zooplankton group that demonstrate resistance to microcystin-LR. Copepod resistance varies by species, but ranges from an LC50 of 270 μg L^-1^ to 1550 μg L^-1^ [30, 34, 35]. Although the LC_50_ calculations from these studies may not be directly comparable to this study due to the differences in conditions tested and methods used, the orders of magnitude difference in the LC_50_ between *B*. *longirostris* and copepods and cladocerans still shows a notable difference in toxicity. In this case, the resistance of copepods refers to the population’s ability to survive by 50% microcystin-LR concentrations of 270–1550 μg L^-1^. Copepods, while showing a greater survival to microcystin-LR than *B*. *longirostris* (LC50 of 26.3 μg L^-1^), are known for their chemosensory avoidance of *M*. *aeruginosa* cells, unlike *B*. *longirostris* [38, 39]. Therefore, while copepods are very resistant to microcystin-LR, the likelihood that they would willingly ingest toxic *M*. *aeruginosa* cells is low, but they could still be exposed to lysed microcystin-LR in the environment. In comparison, *B*. *longirostris* actually feeds on *M*. *aeruginosa* blooms regardless of poor nutritional value or algal morphology (unicellular or colonial) [38, 39]. *B*. *longirostris*, therefore, may have a higher probability of being exposed to microcystin-LR due to the *M*. *aeruginosa* cells that they ingest and microcystin-LR dissolved in the surrounding water column.

**Table 1:** Common zooplankton species and their corresponding LC_50_ values when exposed to dissolved microcystin-LR. Dashes indicate value not reported.

Zooplankton	Species	LC_50_ (μg L^-1^)	Duration	Temperature	Reference
**Cladoceran**	***B*. *longirostris***	**26.3**	**48 hours**	**25**°**C**	**This study**
Cladoceran	*Daphnia pulex*	9600	48 hours	—	DeMott et al., 1991
Cladoceran	*D*. *hyalina*	11600	48 hours	—	DeMott et al., 1991
Cladoceran	*D*. *pulicaria*	21400	48 hours	—	DeMott et al., 1991
Copepod	*Eurytemora affinis*	1550	48 hours	18°C	Ger et al., 2009
Copepod	*Pseudodiaptomus forbesi*	520	48 hours	24°C	Ger et al., 2009
Copepod	*E*. *affinis*	270	48 hours	13.5°C	Reinikainen et al., 2002
Copepod	*Diaptomus birgei*	450	48 hours	—	DeMott et al., 1991

Additionally, in this study, susceptibility of *B*. *longirostris* to microcystin-LR toxicity increased as a function of temperature. This finding indicates that increasing temperatures within the environment increased the susceptibility of *B*. *longirostris* to microcystin-LR. This is also seen in other studies that have exposed zooplankton to temperature and microcystin-LR or toxic *M*. *aeruginosa* cells. *D*. *pulex* was shown to have increased mortality when exposed to higher temperatures [31] and exposure rotifers to *M*. *aeruginosa* at different temperatures showed that as temperature and concentration of cells increased, survivability and reproduction of rotifers decreased [32]. In a study exposing *B*. *longirostris* to *M*. *aeruginosa* cells at different temperatures, Jiang et al. estimated the effects of temperature on toxicity and found that at 20°C *B*. *longirostris* exhibited a higher growth rate than at 28°C, indicating that higher temperatures may negatively affect population growth [33]. In this current study, *B*. *longirostris* experienced a greater mortality at 27°C, compared to 20°C (Figs 3 and 4). With the addition of microcystin-LR at 27°C, *B*. *longirostris* mortality increased significantly (Fig 3). Comparatively, during exposure to toxic *M*. *aeruginosa* cells, *B*. *longirostris* experienced a greater mortality at 28°C than at 20°C [33]. This finding is supported by the data from this study: at 27°C, *B*. *longirostris* had a significantly higher mortality to microcystin-LR (26.3 μg L^-1^) than at 25°C. From 25°C to 27°C, temperature plus toxin mortality increased by nearly 30% (Fig 3). This demonstrates that regardless of how *B*. *longirostris* are exposed, whether by direct exposure to microcystin-LR or to *M*. *aeruginosa* cells [33], they experience a greater mortality at higher temperatures. It is important to note, while the concentration of microcystin-LR in our experiment (26.3 μg L^-1^) did not change between the 25°C and 27°C treatments based on ELISA analysis, some studies have shown that microcystin degrades at temperatures above 30°C [40, 41]. Additionally, while the initial acclimation time for the organisms prior to the experiment may have added additional stress, making them susceptible to higher temperatures, they were all treated similarly and results of the experiments were reproducible, suggesting that the combination of temperature and microcystin-LR was an important factor in toxicity.

It was also noted that *B*. *longirostris* populations were more or less susceptible to microcystin-LR temperature treatments depending on what time of year they were collected and exposed to microcystin-LR. *B*. *longirostris* collected in April 2017 had a temperature plus toxin mortality approximately 33% higher than the temperature plus toxin mortality for *B*. *longirostris* collected in June 2016 at 25°C. In temperature only controls in which no microcystin-LR was added, both populations were exposed to 25°C under the same conditions and showed no significant difference in mortality, indicating that this was not a response to temperature stress alone. We suggest, in turn, that this may be a seasonal effect between the two different populations of *B*. *longirostris* used in this study (April 2017, 15–25°C and June 2016, 25–35°C), because the respective temperature plus toxin mortality at 25°C was not identical for both populations. While demonstrating seasonal effects and adaptation to microcystin-LR exposure is beyond the scope of this study, the results presented here suggest it is possible that microcystin-LR toxicity may not be static within all *Bosmina* spp. populations. There may be a potential for *B*. *longirostris* to adapt to microcystin-LR concentrations as they persist in the environment, additionally altering the LC_50_. Microcystin-LR is only present in the Chowan River seasonally with *M*. *aeruginosa* blooms from June to September [13]. *B*. *longirostris* collected in June 2016 may have been previously exposed to microcystin-LR from a *M*. *aeruginosa* bloom and adapted to the toxin as well as the higher summer temperatures. This suggests that *B*. *longirostris* collected in April 2017 may be less tolerant to microcystin-LR exposure, since it occurs in the Chowan River after they were collected. Evidence has been shown that other cladocerans, such as *Daphnia* spp., can adapt to continuous toxic *M*. *aeruginosa* cell exposure [42–46]. Therefore, additional research is warranted to determine if seasonal acclimation or adaptation to microcystin-LR is a possibility for *B*. *longirostris* and if it is consistently seen across all coastal systems in addition to the Chowan River.

Based on previous literature, *B*. *longirostris* were expected to be largely resistant to toxic strains of *M*. *aeruginosa*. Fulton conducted a prominent study exposing *B*. *longirostris* to toxic *M*. *aeruginosa* cells and demonstrated that *B*. *longirostris*, while gaining no nutritional value from the toxic *M*. *aeruginosa* cells, did not actively avoid ingesting them and were resistant (post ingestion) to these cells [20]. On the contrary, this study found that *B*. *longirostris* were very susceptible to microcystin-LR compared to other zooplankton, (Table 1). It is unclear which specific toxins, such as microcystins or anatoxins, or what the concentration of the toxins present (either intracellular or in the medium) were used by Fulton [20], which could explain the discrepancies with the results of this study. Additionally, mode of toxin delivery could be responsible for the difference in resistance seen. In previous studies, *B*. *longirostris* ingested *M*. *aeruginosa* cells [18, 20, 38], whereas in this study, mode of delivery was microcystin-LR dissolved directly in the water column and filtered by *B*. *longirostris*. To the author’s knowledge, this is the first study that directly quantifies the LC_50_ of microcystin-LR to *B*. *longirostris* when exposed to dissolved microcystin-LR in the water column, rather than ingested.

Because dissolved toxins in the water column is an important mechanism through which exposure can occur, this study advances our understanding of how *B*. *longirostris* may respond in the environment, such as the Chowan River, and how it may ultimately affect the food web. While these results do not directly simulate all environmental conditions and have limitations, they provide a framework for predicting what may occur. Possible removal of *B*. *longirostris* from the Chowan River food web (due to increased temperatures from climate change and microcystin-LR concentrations above the LC_50_ (26.3 μg L^-1^) for the species) effectively places pressure on anadromous fisheries along the Southeastern coastline, potentially decreasing the economic output of the fishery and severely impacting ecosystem functions. Findings from this study also have potential large-scale implications with respect to increasing global sea surface temperatures due to climate change on coastal marine food webs. Increasing water temperatures have been linked to the expansion and increased intensity of cyanobacterial blooms coastally, like *Microcystis* spp. [6, 7, 47–49]. Consistently warmer temperatures could also lead to a prevalence of higher microcystin-LR concentration harmful algal blooms [6, 25, 49]. Current projections for water temperature rise for the Southeast region of the U.S. continental shelf is 2.1±0.1°C to 3.4±0.1°C by 2100 (predicted from four different climate change models) [50–54]. Longer blooms with higher toxin concentrations are also particularly harmful for aquatic life [25]. While *B*. *longirostris* have long coexisted with *M*. *aeruginosa* blooms, and while some studies suggest they are associated with higher microcystin concentrations [26], increased temperatures and higher microcystin-LR concentrations could increase susceptibility to microcystin-LR. Results from this study can be used as a critical first step in informing environmental managers concerned about the spread of *M*. *aeruginosa* and microcystin-LR toxins throughout the Southeast region of the U. S. and globally in conjunction with climate change.

This study helps to clarify the relationship between the specific effects of microcystin-LR when dissolved in the water column and toxicity response by *B*. *longirostris* under varying temperatures. While additional field-based studies are necessary to link these results to *in situ* responses of *B*. *longirostris* to harmful algal blooms under increasing temperatures in the Chowan River, the results of this study are a first step in the process of identifying the combined effects of both temperature and microcystin-LR concentrations on *B*. *longirostris* populations.

## Supporting information

S1 Dataset Ethanol toxicity to *Bosmina longirostris*. *B*. *longirostris* organism mortality after exposure to increasing amounts of 100% ethanol in 100 mL filtered Chowan River water after 24 hrs.
(XLSX)

S2 Dataset Microcystin-LR toxicity dose-response to *Bosmina longirostris*. *B*. *longirostris* organism percent mortality to microcystin-LR concentrations 0.005 μg L^-1^ to 100 μg L^-1^ at 25°C after 48 hrs exposure.
(XLSX)

S3 Dataset Probit dose-response analysis. Empirical probits as a function of log-microcystin-LR dose to *Bosmina longirostris* mortality.
(XLSX)

S4 Dataset High temperature and microcystin-LR toxicity to *Bosmina longirostris*. *B*. *longirostris* organism mortality after exposure to 25°C to 35°C and microcystin-LR at 26.3 μg L^-1^ for 48 hrs.
(XLSX)

S5 Dataset Low temperature and microcystin-LR toxicity to *Bosmina longirostris*. *B*. *longirostris* organism mortality after exposure to 15°C to 25°C and microcystin-LR at 26.3 μg L^-1^ for 48 hrs.
(XLSX)
